# Multigene Phylogeny and Pathogenicity Trials Revealed *Alternaria alternata* as the Causal Agent of Black Spot Disease and Seedling Wilt of Pecan (*Carya illinoinensis*) in South Africa

**DOI:** 10.3390/pathogens12050672

**Published:** 2023-05-02

**Authors:** Conrad Chibunna Achilonu, Gert Johannes Marais, Soumya Ghosh, Marieka Gryzenhout

**Affiliations:** 1 Department of Plant Sciences, Division of Plant Pathology, Faculty of Natural and Agricultural Sciences, University of the Free State, Bloemfontein 9300, Free State, South Africa; 2 Department of Genetics, Faculty of Natural and Agricultural Sciences, University of the Free State, Bloemfontein 9300, Free State, South Africa

**Keywords:** *Alternaria alternata*, black spot disease, multigene phylogeny, pathogenicity test, pecans (*Carya illinoinensis*)

## Abstract

The pecan (*Carya illinoinensis*) industry in South Africa is growing rapidly, and it is becoming increasingly crucial to understand the risks posed to pecans by fungal pathogens. Black spots on leaves, shoots, and nuts in shucks caused by *Alternaria* species have been observed since 2014 in the Hartswater region of the Northern Cape Province of South Africa. Species of *Alternaria* include some of the most ubiquitous plant pathogens on earth. The aim of this study was to use molecular techniques to identify the causative agents of Alternaria black spot and seedling wilt isolated from major South African pecan-production areas. Symptomatic and non-symptomatic pecan plant organs (leaves, shoots, and nuts-in-shucks) were collected from pecan orchards, representing the six major production regions in South Africa. Thirty *Alternaria* isolates were retrieved from the sampled tissues using Potato Dextrose Agar (PDA) culture media and molecular identification was conducted. The phylogeny of multi-locus DNA sequences of *Gapdh*, *Rpb2*, *Tef1*, and *Alt a 1* genes revealed that the isolates were all members of *Alternaria alternata sensu stricto,* forming part of the *Alternaria alternata* species complex. The virulence of six *A. alternata* isolates were tested on detached nuts of Wichita and Ukulinga cultivars, respectively, as well as detached leaves of Wichita. The *A. alternata* isolates were also evaluated for their ability to cause seedling wilt in Wichita. The results differed significantly between wounded and unwounded nuts of both cultivars, but not between the cultivars. Similarly, the disease lesions on the wounded detached leaves were significantly different in size from the unwounded leaves. The seedling tests confirmed that *A. alternata* is pathogenic and that *A. alternata* causes black spot disease and seedling wilt of pecans. This study is one of the first documentations of Alternaria black spot disease of pecan trees and its widespread occurrence in South Africa.

## 1. Introduction

*Carya illinoinensis*, better known as the pecan tree, is a species of hickory that is indigenous to northern Mexico and the southern United States of America (USA), and is distributed along the Mississippi River, which extends from northern Illinois and south-east Iowa to the Gulf Coast, with most of the species’ diversity found in Texas and the state of Oklahoma [1]. The genus *Carya* belongs to the Juglandaceae [1] family, and comprises 20 species [1]. It is divided into two groups (native and improved cultivars), namely the *Carya* sect. *Sinocarya* and *Carya* sect. *Carya* [2,3]. Among these species, six are native to China, Indonesia, and India. Twelve species are native to the USA, four are found in Mexico, and two are from Canada. Improved cultivars and species were introduced worldwide for the production of high-quality nuts at the commercial level in countries such as Argentina, Australia, Brazil, China, and South Africa [4]. The global production and export of pecans are on the rise, particularly in South Africa [5]. The USA is the largest leading producer of pecan nuts with about 50% of the world’s production, followed by Mexico, South Africa, and Australia [6]. South Africa produces between 15,500 and 20,000 tons per year and exports about 90% of its pecan nuts to China due to the high value per metric tons of pecan nuts, and 8% to Europe, with the remaining 2% used in the domestic market [5,7]. Pecan nuts are seen as a luxury and are not readily affordable for the average South African household; hence, production is primarily aimed at exports. In South Africa, the pecan industry is growing at a fast rate, and the nuts are commercially in demand in the pecan markets around the world [8]. Moreover, there is a recently increased recognition of the value of pecan nuts and a realisation of their income-generating potential through trade, which is significant for the economic growth of the industry.

Favourable environmental conditions such as climate, soil, pest- and disease-free environments [9], and essential nutrients [10] are crucial to grow healthy pecans and produce high yields of the nuts. However, pecan trees in South Africa are often planted on previously uncultivated lands, or lands where other crops have been planted in the past [11]. This often allows for the establishment of new interactions between plants and living organisms, such as insects, smaller animals, and microorganisms [12,13]. This also leads to the continuous development of diseases that do not exist on pecans in other producing countries. One such example is the attack on pecans by the phytopathogenic fungal genus *Alternaria* [14].

*Alternaria* species are common, able to grow under a large range of temperatures [15], and associated with a variety of substrates such as animals, humans, plants, seeds, and soils [16]. *Alternaria* comprises nearly 300 species that occur worldwide [17,18,19] as either plant-pathogenic or saprotrophic organisms [20]. Some of these species have caused economic losses on many crops worldwide due to their ability to cause diseases that impact plant health or yield [21]. Previously, species of *Alternaria* were often characterised based on host associations [16], and known to infect over 400 host plants, causing various disease symptoms [22]. *Alternaria alternata* is the most reported species of *Alternaria* [23]. It is an important plant pathogen known to cause Alternaria black spot (ABS) and seedling blight on plants [24,25]. The infected plant organs develop symptoms such as small, circular black spots that eventually progress to large black lesions [26]. There are several reports of severe global outbreaks of the pathogen, causing disease for various plants and crops such as black chokeberry (*Aronia melanocarpa*) in Korea [27], melon (*Cucumis melo*) in China [28], pomegranate (*Punica granatum*) in Spain [29], Japanese pear (*Pyrus pyrifolia*) in France [30], pear black spot (*Pyrus bretchneideri*) in China [31], and rubber tree (*Ficus elastica*) in China [24]. Furthermore, *A. alternata* was reported to cause severe leaf blight on potatoes (*Solanum tuberosum*) in South Africa [32]. The pathogen is also associated with the damping-off of seedings such as cypress (*Cupressaceae*), green ash (*Fraxinus pennsylvanica*), honey locust (*Gleditsia triacanthos*), bur oak (*Quercus macrocarpa*), northern catalpa (*Catalpa speciosa*), and hackberry (*Celtis occidentalis*) in tree nurseries [33].

**Figure 1:**
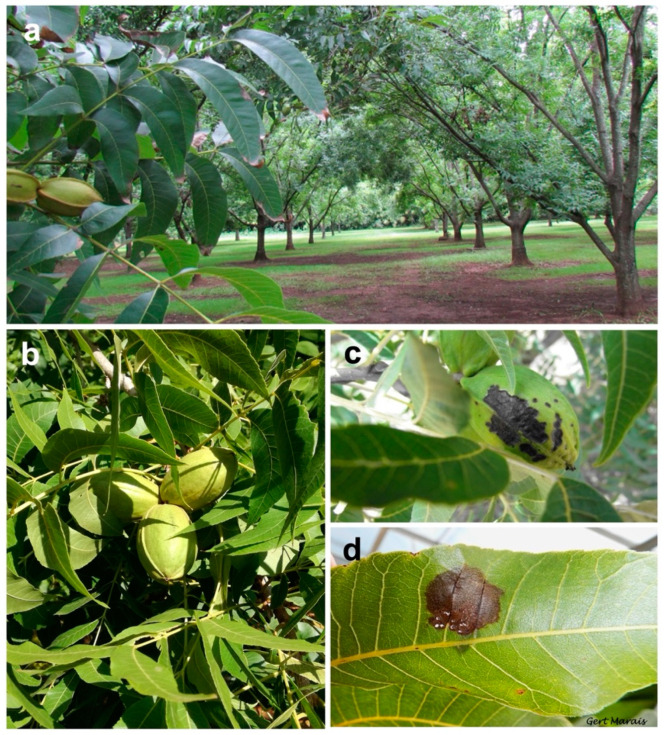
Symptomatic and non-symptomatic pecans (*Carya illinoinensis*) in the field. (**a**) Pecan orchard. (**b**) Non-symptomatic pecan leaves and nuts in shucks. (**c**,**d**) Black spot symptoms on pecan leaves and nuts (shucks) [36].

In South Africa, ABS has recently become one of the more common fungal foliar and nut diseases noticed on pecans across South Africa [34]. The symptoms of the disease on pecans are similar to symptoms on other crops. Under critical conditions, lesions coalesce to form large areas of necrosis, resulting in the defoliation of leaves and premature shedding of nuts and elongated superficial black lesions on shoots (Figure 1). Furthermore, wilt or overall decline of pecan trees is gradually becoming a potential limitation to the development and production of pecan nuts [35]. Affected pecan trees tend to show stunting, followed by wilted leaves, and eventually death of those severely infected by a diversity of fungi, of which *A. alternata* could be one. Therefore, it is of crucial importance to properly identify this pathogen at a molecular level and to test its virulence, since *A. alternata’s* association with ABS disease and wilting in South African pecan has not yet been investigated.

DNA-based multigene phylogenetic analyses have been used to delineate the complex taxonomic classification of *Alternaria* species [19,22]. The taxonomy of *A. alternata* section was recently revised based on 10 gene regions, namely the nuclear small subunit (*SSU*) rRNA, large subunit (*LSU*) rRNA, internal transcribed spacer (*ITS*), glyceraldehyde-3-phosphate dehydrogenase (*Gapdh*), RNA polymerase II 2nd largest subunit (*Rpb2*), translation elongation factor 1-α (*Tef1*), *Alternaria* major allergen (*Alt a1*), endopolygalacturonase (*EndoPG*), anonymous gene region (*OPA10-2*), and eukaryotic orthologous group (*KOG*) [37]. Several studies have shown that multigene phylogenetic identification can classify or segregate *A. alternata,* known to cause black spot disease. For instance, Aung et al. [38] reported the first case of small-spored *A. alternata* associated with Koerle pear (*Pyrus sinkiangensis*) in Korea based on a multigene phylogeny of *Gapdh, Rpb2*, and *Alt a1* genes. Chen et al. [39] used the multigene phylogenetic analyses of *ITS*, *Gapdh*, and β-tubulin genes to characterise *A. alternata*, a causal agent of black spot tea plant (*Camellia sinensis*), in the Chongqing district of China. Kgatle et al. [21] recently showed that the multi-locus phylogeny of *Alt a1, Rbp2, Gapdh*, *Tef1*, and *ITS* gene regions successfully identified *A. alternata,* causing leaf blight on sunflower (*Helianthus annuus*) in South Africa.

In the present study, we aimed to investigate which *Alternaria* species are associated with the symptoms of ABS disease on pecan in South Africa. The identification of *Alternaria* species was based on a multi-locus sequence analysis of the *Gapdh*, *Rpb2*, *Tef1*, and *Alt a1* gene regions as previously reported [37]. The present study further tested the pathogenicity of *Alternaria* isolates and their ability to cause black spot and seedling wilt in pecans.

## 2. Materials and Methods

### 2.1. Sample Collection and Isolation of Alternaria

**Figure 2:**
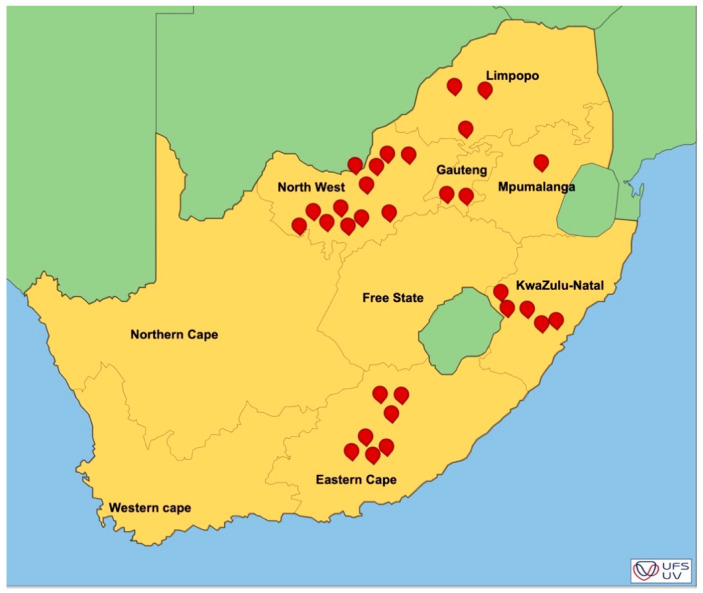
Map depicting pecan orchard locations (red beacons) where *Alternaria alternata* isolates were sampled from six provinces in South Africa.

Symptomatic and non-symptomatic pecan plant organs (leaves, shoots, and nuts-in-shucks) were collected from various commercial pecan orchards (Figure 2). These locations include the Eastern Cape, Gauteng, Kwazulu-Natal, Limpopo, Mpumalanga, and north-west provinces of South Africa from 2017 to 2019 in the spring and summer seasons (Figure 2 and Supplementary Materials: Table S1). Samples of the healthy and diseased pecan leaves, nuts, and shoots were placed in separate paper bags to avoid desiccation and cross-contamination, kept in an ice box, and transported to the laboratory.

The non-symptomatic plant tissues and lesion areas were cut (5 × 5 mm) from individual plant material. Only pieces from the edge of lesions were used. Pieces were surface-sterilised with 70% ethanol for 30 s, rinsed 3 times with sterilised water (distilled water—dH_2_O), and dried for 5 min on a sterile bench and transferred to a Petri dish (90 mm) (Lasec, Bloemfontein, South Africa) containing potato dextrose agar (PDA: potato 200 g, dextrose 20 g, agar 16 g/L) (Merck-Millipore, Pretoria, South Africa) amended with chloramphenicol (0.01 g/L) (Biologica Pharmaceuticals, Pretoria). The Petri dishes were incubated at 25 °C under 12 h alternating cycles of near-ultraviolet (NUV) (360 nm wavelength) light and darkness for 5 days. *Alternaria* cultures that grew from the original plates were transferred to fresh PDA plates and separated into morphotypes based on colour and texture differences [40]. The Petri dishes were incubated in a Labcon LTGC-M40 incubator (Labcon, Gauteng, South Africa) at 25 ± 1 °C under 12 h alternating cycles of near-ultraviolet (NUV) (360 nm wavelength) light and darkness for 7 days. Cultures were purified by performing a single hyphal tip removal and plated onto PDA media under incubator conditions mentioned above. All the representative cultures were transferred to 2 mL cryogenic tubes (Merck-Millipore, Pretoria, South Africa) containing 0.85% saline (sodium chloride dissolved in distilled water) −15% glycerol solution and stored at −80 °C until required. The cultures were subsequently deposited into the National Collection of Fungi at the Agricultural Research Council-Plant Protection Research Institute (ARC-PPRI), Pretoria, South Africa.

### 2.2. Morphological Identification

Morphological identification was evaluated based on colony characteristics, conidial sizes, and the presence of catenulate conidia [18]. *Alternaria* isolates were cultured on petri plates containing half-strength PDA (19.5 g potato dextrose agar (Neogen Corporation, UK), 8 g agar (Biolab Millipore, South Africa), dissolved in 1L distilled water and autoclaved for 7 days at 25 ± 1 °C under 12 h alternating cycles of NUV light and darkness. Measurements of conidia and conidiophores were conducted after 7 days. A representative sample was mounted on a glass microscope slide and 5% lactophenol solution. Ten measurements of each structure were recorded at 10 to 100× magnification using an Olympus BX53F microscope (Olympus Corporation, Tokyo, Japan). Colony pigmentation was compared to the colour chart of Rayner [41].

### 2.3. DNA Sequencing

Genomic DNA (gDNA) was extracted from scraped, 14-day-old cultures of 30 *Alternaria* isolates representative of the various morphotypes (Table S1). This was carried out using the ZR Quick-DNA Fungal/Bacterial Microprep™ Kit (Zymo Research, CA, USA). The DNA concentration and purity were determined with a NanoDrop Lite ND-2000 spectrophotometer (Thermo Fisher Scientific, MA, USA).

Polymerase chain reactions (PCR) were performed separately for four gene regions (*Alt a1*, *Gapdh*, *Rpb2* and *Tef1*) and sequenced using primers listed in Table 1. Each PCR reaction (50 μL) consisted of 50–100 ng of template DNA, 0.3 μM of each primer, 2.5 mM MgCl_2_, 0.3 mM of each dNTP, and 1 U KAPA HiFi HotStart DNA Polymerase (Kapa Biosystems-Roche, Basel, Switzerland). The T100TM Thermal Cycler conditions (Bio-Rad, CA, USA) for PCR amplification entailed an initial denaturation step of 3 min at 95 °C followed by 24 cycles of 20 s at 98 °C, 30 secs annealing step at 58.2 °C (*Gapdh* and *Rpb2*), 61.3 °C (*Tef1*), and 63.3 °C (*Alt a1*), 60 s at 72 °C and a final elongation step of 3 min at 72 °C. The PCR products stained with GelRed nucleic acid stain (Thermo Fisher Scientific), were examined on a 2% agarose gel electrophoresis, visualised under UV light “Gel Doc EZ Gel Documentation System” (Bio-Rad). The PCR products were purified using the Zymo DNA Clean & Concentrator Kit (Zymo Research).

The purified PCR amplicons for all the genes were sequenced in both primer directions using the BigDye Terminator v.3.1 kit (Applied Biosystems, CA, USA). Reactions contained 1 × BigDye Terminator Premix, 3.2 µM PCR primer, 1 × Sequencing buffer, and 3 µL PCR amplicon, in a total volume of 10 µL. The conditions for the thermal cycler consisted of an initial denaturation step at 96 °C for 1 min, followed by 25 cycles of 96 °C for 10 s, 50 °C for 5 s, and 60 °C for 4 min. Cycle sequencing products were purified using the ZR DNA Sequencing Clean-Up^TM^ Kit (Zymo Research). The purified sequencing products were analysed with an ABI 3500xl Genetic Analyzer (Applied Biosystems), using standard protocols.

### 2.4. Phylogenetic Analyses

The PCR amplicons of the four genes were 980, 860, 1020, and 720 base pairs (bp) for *Alt a1*, *Gapdh*, *Rpb2*, and *Tef1,* respectively (Table 1). Forward and reverse sequence reads were combined in Geneious v.11.1.5 [46]. DNA sequence datasets of Woudenberg et al. [47] were imported from TreeBASE (https://www.treebase.org/treebase-web/home.html accessed on 15 October 2019) and used for phylogenetic analysis. The multi-locus gene sequences were aligned using MAFFT v.7 [48] and manually adjusted using MEGA X. v.10.1 [49] where necessary. JModelTest v.2.1.10 was employed to estimate the best nucleotide substitution model for both the individual gene and combined datasets [50].

For phylogenetic analyses, Bayesian inference (BI) analyses were performed for both the individual gene sequence and the combined aligned datasets. Bayesian inference (BI) analysis was performed with MrBayes v.3.1.2 [51,52]. The batch scripts (nexus file) for the combined dataset included two parallel runs, four chains each (three heated, one cold), temperature 0.2, substitution models: HKY+G for *Tef1*-(partition 1), and TNe+G for *Alt-a1*, *Gapdh*, *Rpb2*-(partitions 2 to 4). All parameters used for the single-gene phylogenies were the same, except for the substitution models (GTR+G for *Alt a1* and gapdh and K2P+G for *Tef1* and *Rpb2*). The Markov Chain Monte Carlo (MCMC) analysis used four chains and started from a random tree topology. The sample frequency was set at 100 and the temperature value of the heated chain was 0.1. The Markov Chain Monte Carlo (MCMC) chains were run for 1,000,000 generations with sampling frequency of one tree every 1000 iterations. The temperature value of the heated chain was (2), and the run stopped when the average standard deviation of split frequencies fell below (0.02). Furthermore, the burn-in was set to 25% once the likelihood values were stationary. A maximum-likelihood (ML) analysis, including 1000 bootstraps, was performed on the combined aligned dataset using raxmlGUI v.2.0 [53,54]. Finally, the generated phylogenetic tree topologies were adjusted and viewed using FigTree v.1.4.4 [55].

### 2.5. Pathogenicity Tests

Wichita and Ukulinga cultivars (cv.) of pecans were used for these bioassays. The detached pecan nuts used included Wichita and Ukulinga cv. obtained from Hartswater (27°50′33.5″ S 24°46′10.5″ E), Northern Cape, South Africa. The pathogenicity of the *A. alternata* isolates used in the detached nut assays was performed using a modified method by Saaiman [56]. Detached nuts were used to determine the ability of the *A. alternata* isolates to penetrate through the surface of healthy unwounded and wounded nuts and cause lesions. However, wound openings were tested as it has been shown that a wound creates an opportunity for infection [56].

Six selected *A. alternata* isolates (CGJM3006, CGJM3078, CGJM3103, CGJM3136, CGJM3137, CGJM3142), identified by multi-locus sequence phylogeny, were used for pathogenicity tests and they were grown on half-strength PDA culture medium at 25 ± 1 °C on 12 h alternating NUV light and dark cycles. The bioassay was performed in three containers (17 × 260 × 88 mm) according to the following procedure: a total of 10 Wichita and Ukulinga nuts were placed in their respective containers and arranged 20 mm apart in a sequence of five nuts per row. The nuts were individually attached to the plastic grids using a Blu-Tack reusable adhesive, and the grids with the attached nuts were placed on two opened Petri dishes containing sterile water (dH_2_O) to maintain high relative humidity (±85%). On each row in the container, five nuts were wounded (pricked) using a sterilised hypodermic needle (one wound per nut) and the other five were not wounded. Then, an agar plug (4 × 4 mm) taken from each growing culture was placed against the wounded and unwounded nut surface and covered with parafilm. In a separate container, PDA plugs (4 × 4 mm) without fungal mycelia were used as controls. All treatments were performed in triplicate, with one container per replicate. The containers were incubated at 25 ± 1 °C on 12 h light-and-dark cycles. The developing black spot lesions were monitored at 2-day intervals to prevent desiccation and evaluated 14 days post-inoculation (p.i.). To corroborate Koch’s postulates, sections of the diseased nuts per treatment were placed onto half strength PDA medium and incubated at 25 ± 1 °C with 12 h alternating NUV light and dark cycles until *A. alternata*-like isolates were observed. These isolates were examined for the presence of spores to confirm the identity of the fungi.

Pathogenicity tests for detached leaves were conducted with the method described by Giri et al. [57]. Only fresh leaves of cv. Wichita were available, and not cv. Ukulinga, during pathogenicity testing. Twenty-four pecan compound leaves were obtained from three-year-old healthy trees grown in a greenhouse at the University of the Free State, South Africa. The leaves were surface-sterilised using the previously described procedure. Each leaf was placed in a container (170 × 260 × 88 mm). The petiole end was inserted into a 10 mL McCartney bottle (Lasec, South Africa) containing sterile water (dH_2_O) to prevent desiccation. The bottle was sealed and attached to the plastic grids with Blu-Tack adhesive press-stick. Three leaflets per container were wounded in the middle using a sterile needle, while the opposite three were not wounded. The same six *A. alternata* isolates were used as the inocula. The inoculation was performed by placing a 4 × 4 mm agar plug of a growing mycelia culture on the respective wounded and unwounded sites, and each treatment was covered with adhesive tape. The PDA plugs (4 × 4 mm) with no fungal mycelia were used as controls. All the treatments were conducted in duplicate, with one container per replicate. The inoculated leaflets were placed in humidity chambers and incubated in a Labcon LTGC-M40 incubator (Labcon) at 25 ± 1 °C on 12 h alternating NUV light and dark cycles. The developing symptoms on the inoculated leaflets were observed 14 days post-inoculation (p.i.). Koch’s postulates were corroborated according to the procedure described above for nut inoculations.

Evaluation of the *A. alternata* isolate infections for detached nut and leaf bioassays was analysed as described by Mohamed-Azni et al. [58] and Gryzenhout et al. [59]. The leaf and nut lesions were scored 14 days post-inoculation (dpi) using a five-point rating system: 0 = No lesion, 1 = small lesion < 1 mm (1–25% mild infection rate), 2 = intermediate lesions from >1 to 3.5 mm (26–50% moderate infection rate), 3 = large lesions from 3.6 to 5.9 mm (51–75% severe infection rate), 4 = Lesions covering the nut and leaf defoliation > 6 mm in diameter (76–100% highly severe infection rate). The diameter (mm) of the lesions and no lesion (control) were measured using ImageJ v.1.53 software [60]. Two orthogonal diameter measurements were calibrated as the scale standard of the images. Lesion diameters were determined at 0° and 90° measurements, and the mean diameter values were used accordingly.

Three-way analysis of variance (ANOVA) of the disease severity data for detached nuts was performed using R v.4.1.0 [61] within R-Studio v.1.3.959 [62] to determine the significant effects of the representative *A. alternata* isolate, treatment (wounded and unwounded), and host tissue (cultivars). A two-way ANOVA was carried out to determine the significance of the *A. alternata* isolate and treatment (wounded and unwounded) for the leaf assays. Means were compared at a significant level of 0.05 using Fisher’s least significant differences (LSD) test function from the ‘agricolae’ [63] and ‘doebioresearch’ (Analysis of Factorial Randomised Block Design for 3 factors) packages [64]. Graphs were plotted with the estimated means using Microsoft^®^ Excel 2020 v.16.39. to visualise the significant and non-significant factors.

### 2.6. Seedling Wilt Assay

Pathogenicity tests were conducted to validate whether the same selected six *A. alternata* isolates can cause seedling wilt of 6-week-old pecan seedlings of cv. Wichita. The seedlings were grown for 6 weeks in 300 × 220 mm plastic pots containing sterilised and fertilised soil potting mediums (in 1:1 ratio, *w*/*w*). Three replicate pots per treatment were prepared. Spore suspensions of the same isolates used previously were prepared by adding dislodged conidia obtained from 14-day-old cultures and were placed into 45 mL potato dextrose broth (PDB: potato 4 g/L, dextrose 20 g/L) (Thermo Fisher Scientific). The solutions were placed in a shake-incubator (Apex-Scientific, Gauteng, South Africa) for 21 days at 25 ± 1 °C for spore growth. The spore concentrations were determined using a hemocytometer [65] and adjusted to 1 × 10^6^ spores/mL to suppress seedlings.

A channel, 5 mm wide and 120 mm deep, was created in the soil close to each seedling and filled with 45 mL spore suspension. Controls were treated with sterile water (dH_2_O). Three pots per isolate were used for treatment and one pot was used for the control. The inoculated plants were placed in an incubator Labcon LTGC-M 70 incubator (Labcon, Gauteng, South Africa) at 25 ± 1 °C for 12 h light-and-dark cycles and irrigated with sterile water on alternate days.

Wilting symptoms on seedling plants were observed after 56 days. The disease severity of wilt was carried out as evaluated by Nayyar et al. [66], using four-point categorical disease severity scales: 0 = no wilting symptoms, 1 = 25% wilting, 2 = 50% wilting and 3 = 100% wilting and plant death. The disease severity index (DSI) was calculated as ∑ (disease severity scale points × number of plants at each scale point)/(total number of seedlings assessed × disease severity scale of the highest scale point observed) × 100. The DSI data for each treatment were analysed using the previously described ANOVA procedure.

## 3. Results

### 3.1. Morphological Characterization of Alternaria alternata

**Figure 3:**
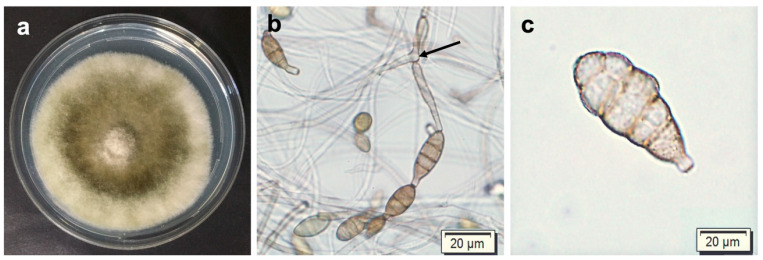
Morphological characteristics of *Alternaria alternata*. (**a**) Mycelial colony of CGJM3006 isolate. (**b**) Arrow depicting conidiophore bearing conidia. (**c**) Conidium. Scale bars: 20 μm.

Isolates showed distinctive morphological characteristics corresponding to those of the *Alternaria alternata* sect. *Alternaria* of Woudenberg et al. [19]. The developing colonies (Figure 3a) produced olive green colorations with grey margins and woolly textures. Conidiophores were 12–50 μm long, ranged from straight to bent, and, in most cases, were branched (Figure 3b). The illustrated conidia were brown and ranged from obpyriform to ellipsoid in shape, with both transverse and longitudinal septa (Figure 3c), 8–15 μm and ranged from 12 to 34 μm in length.

### 3.2. Phylogenetic Analyses

**Figure 4:**
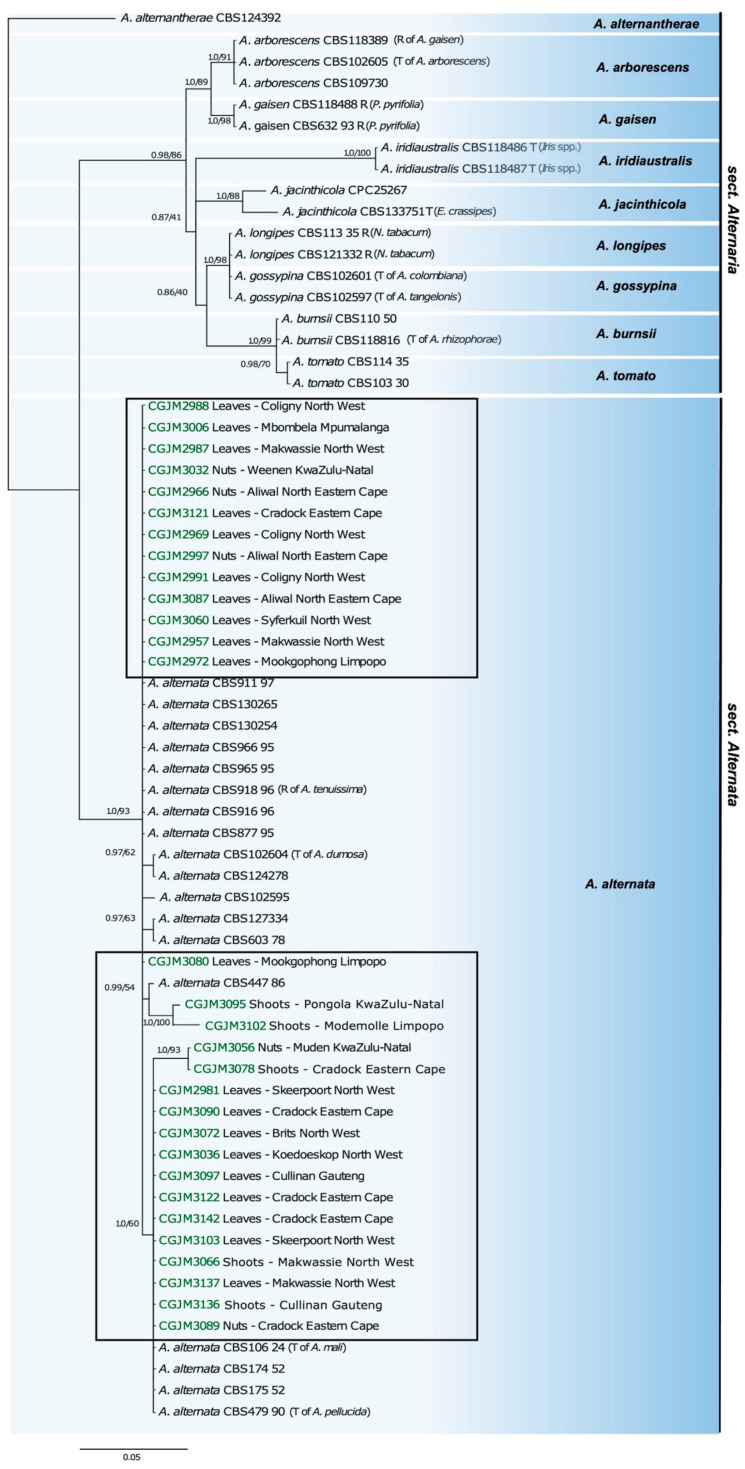
Bayesian inference (BI) tree of *Alternaria* section *Alternata* based on a combined four-gene data set (*Gapdh, Tef1, Rpb2,* and *Alt a1*). The phylogram rooted to *Alternaria alternantherae* (CBS 124392). Bootstrap support (>50) and posterior probability (>0.85) values are indicated on the branch nodes (PP/ML). The *A. alternata* isolates from this study are highlighted in green, with geographical locality and different plant organs indicated. (T): means ex-type isolate, (R): representative isolate, and species names between parentheses refer to the former *Alternaria* species name.

The aligned sequences of the combined dataset generated a total of 1198 characters, while the *Alt a1* dataset resulted in 368 aligned characters, *Gapdh*: 343 aligned characters, *Rpb2*: 249 aligned characters, and *Tef1*: 248 aligned characters. Phylogenetic trees for each gene constructed for the 30 *Alternaria* isolates were always in the *A. alternata* sensu stricto clade (Supplementary Materials Figure S1). The topology of the multigene phylogeny obtained from ML and BI analyses showed similar groupings. Thus, only the BI tree was illustrated (Figure 4), showing 96 in-group and 1 out-group taxa with clades consistent with those in the *Alternaria alternata* sect. *Alternata* of Woudenberg et al. [37]. All the *A. alternata* isolates were clustered within *A. alternata sensu stricto*, with highly supported posterior probability (PP) of 1.0 and an ML bootstrap of 93%. No associations or distinctions between clades, geographical location, or types of plant materials were observed.

### 3.3. Pathogenicity Tests Analyses

**Figure 5:**
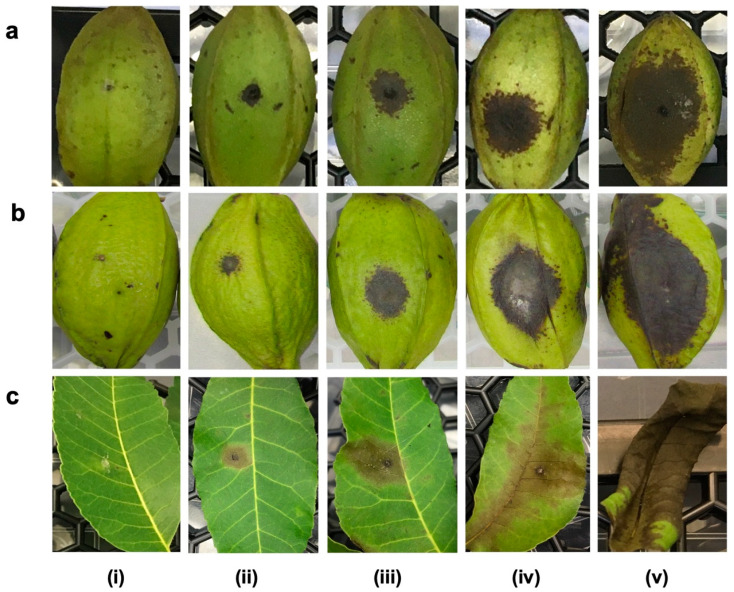
Black spot disease rating used in the pathogenicity tests of *Alternaria alternata* isolates on (**a**) detached nut (shucks) of Wichita cultivar, (**b**) detached nut (shucks) of Ukulinga cultivar and (**c**) detached leaf assays. The rating scale for lesion development on detached nut and leaf was as follows from left to right: (**i**) Rating 0 = no lesion development, (**ii**) 1 = small lesion < 1 mm (1–25% mild infection rate), (**iii**) 2 = intermediate lesions from 1 to 3.5 mm (26–50% moderate infection rate), (**iv**) 3 = large lesions from 3.6 to 5.9 mm (51–75% severe infection rate), (**v**) 4 = lesions covering the nut and leaf defoliation > 6 mm in diameter (76–100% highly severe infection rate).

**Figure 6:**
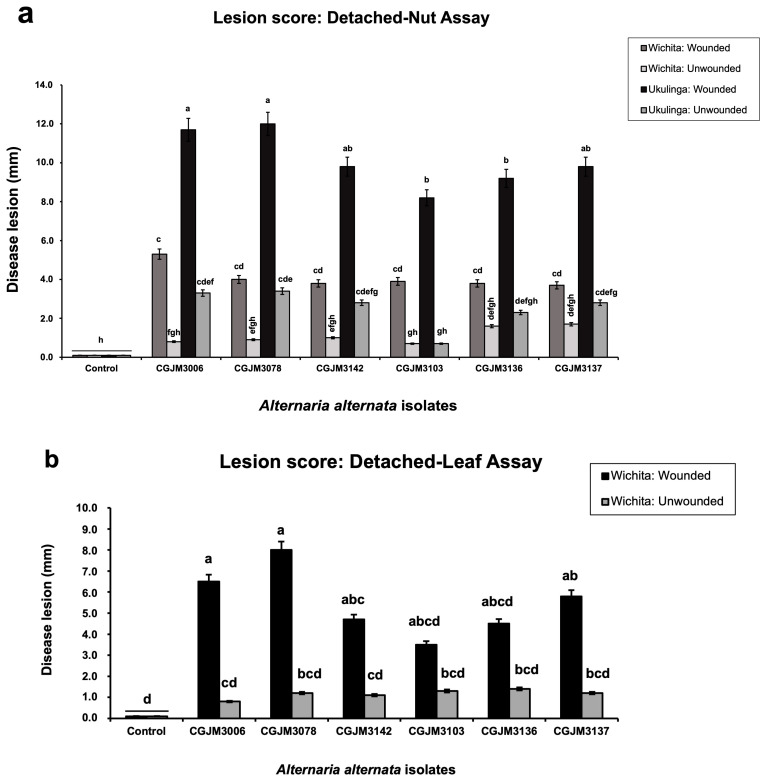
Alternaria black spot lesion from wounded and unwounded nuts and leaves based on the pathogenicity tests of *Alternaria alternata* isolates. (**a**) Wounded and unwounded detached nut assay of Wichita and Ukulinga pecan cultivars. (**b**) Assays on wounded and unwounded pecan leaves of Wichita. Bars represent the standard error, different letters on the bars are significantly different with treatment according to Fisher’s LSD test (*p* < 0.05).

The pathogenicity tests on the detached pecan nuts revealed the same black spot symptoms caused by the tested *A. alternata* isolates as those observed in the field (Figure 5a(i)–b(v)). The lesions initially appeared as small, dark brown colourations that gradually expanded and were occasionally confirmed at 7 dpi, and eventually turned into large lesions at 14 dpi (Figure 5a(ii)–b(v)). The lesions from the replicates (containers) were similar; thus, the scores were combined. The sizes of these lesions ranged categorically from 0 to 4. No lesions formed in the control inoculations (Figure 5a(i)) and the score was zero. The virulence (lesion sizes) of all treated isolates was significantly different (*p* < 0.001) from the controls (Table S2 and Figure 6a).

There were statistical differences (*p* < 0.001) in virulence for each isolate on wounded and unwounded nuts for both cultivars (Table S2 and Figure 6a), as indicated by the different letters (a–h) in each column. This is based on the *A. alternata* isolate, CGJM3103, which was the least virulent, with mild infection when evaluated against unwounded Wichita and Ukulinga nuts, with a mean lesion size of 0.9 mm. Isolates CGJM3078 and CGJM3006 were the most virulent, with severe infection on wounded Ukulinga, with a mean lesion size of 12.0 mm. There were no statistical differences (*p* > 0.001) in the lesion severity of individual isolates between cv. Wichita and cv. Ukulinga. Koch’s postulates were satisfied as described above for the nut inoculations.

The bioassay on the detached pecan leaves after 14 days showed black spot symptoms caused by the tested *A. alternata* isolates with lesion scores from 0 to 4 (Figure 5c(i)–(v)). The lesions initially appeared as small, dark brown colouration that gradually spread and was confirmed at 7 dpi, and eventually expanded into large lesions at 14 dpi (Figure 5c(ii)–(v)). Lesion sizes were not significantly different for all inoculated isolates 14 days after inoculation. The control leaves differed significantly (*p* < 0.001) from the leaves treated with all isolates (Table S3 and Figure 6b). There were significant (*p* < 0.001) differences in the mean disease severity of the lesions formed (mm) between the wounded and unwounded leaves, with the wounded being the most severe. Isolates CGJM3142 and CGJM3006 showed mild infections, as the least virulent, on the unwounded leaves, with a mean lesion size of 1.1 and 0.8 mm, respectively. Isolates CGJM3078 and CGJM3006 showed very severe infection, as the most virulent, when tested on the wounded leaves, with a mean lesion size of 8.0 and 6.5 mm, respectively. Koch’s postulates confirmed that the ABS lesions on the inoculated leaves were caused by the isolates and no other fungal growth or symptoms were observed.

### 3.4. Pathogenicity Analyses of Pecan Seedling Wilt

**Figure 7:**
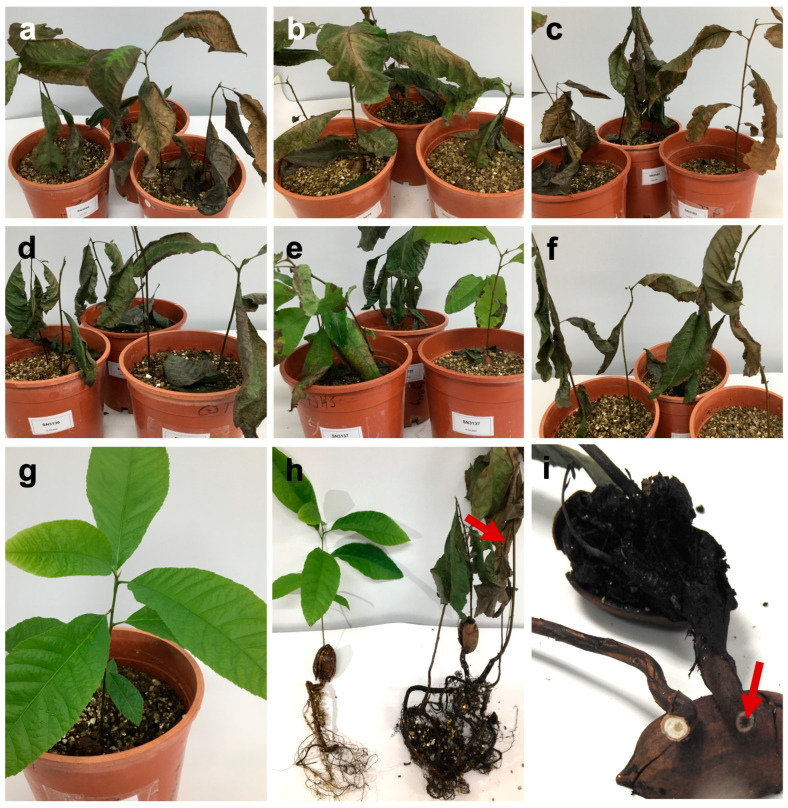
Wilt on pecan seedlings of Wichita cultivar 56 days post inoculation treated with six *Alternaria alternata* isolates: (**a**) CGJM3006, (**b**) CGJM3013, (**c**) CGJM3078, (**d**) CGJM3136, (**e**) CGJM3137, and (**f**) CGJM3142. (**g**) Control, (**h**) Comparison of plants with healthy roots (left) and plants with necrosis (right: indicated with red arrow), and (**i**) root rot (indicated with red arrow).

The Wichita seedlings inoculated with the six individual *A. alternata* isolates displayed wilt symptoms. These isolates resulted in wilting of the leaves, followed by drying and death after 56 days (Figure 7a–f). In most instances, the wilt was associated with the inoculated roots (xylem and phloem) that developed dark brown discolourations and necroses (Figure 7h,i). The statistical data showed mean significant difference (*p* < 0.001) between the control and all the tested isolates. The mean difference was significantly different (*p* < 0.001) between CGJM3137 and the other five isolates (CGJM3006, CGJM3103, CGJM3078, CGJM3136, and CGJM3142) (Table 2), but not significantly different (*p* > 0.001) among these five isolates. CGJM3137, with average (50%) disease severity, was considered less virulent than the other isolates. Isolates CGJM3078 and CGJM3142 (90% severity) were found to be more virulent, with complete wilting of the seedlings. No wilt was observed in the control inoculum seedlings (Figure 7g,h), which retained green leaves and healthy roots compared to those of the wilted plants. The re-isolations during Koch’s postulates confirmed that the isolates described were the cause of wilting (Supplementary Materials Figure S2) and no other fungal growth or symptoms were observed.

## 4. Discussion

This study represents one of the first reports of ABS disease and seedling wilt of pecans in South Africa caused by *A. alternata*. The phylogenetic analyses of the combined gene data set showed that the isolates obtained in this study grouped within *A. alternata* sensu stricto [37]. Wichita and Ukulinga cultivars were selected for pathogenicity tests because they are commonly grown in the main pecan-production areas of South Africa, and they produce quality nuts [67]. The bioassays confirmed the potential virulence and similar lesion phenotypes to those of field observations for all the tested *A. alternata* isolates on detached nuts for Wichita and Ukulinga cultivars, and detached leaves of cv. Wichita. The soil inoculations also resulted in the wilting of pecan seedlings, indicating the ability of the pathogen to affect the health of the root system.

The fungus morphologically showed profuse mycelial growth on the PDA, turning grey–brownish [37,67] in colour, consistent with Woudenberg et al. [19], and spores resembling those of *A. alternata*. Morphology was not the main means of identification, as *Alternaria* isolates could differ morphologically due to the different cultivating conditions and the overlap in the spore sizes of some species [68]. Armitage et al. [69] reported that the morphological characteristics used to delineate species in *Alternaria* sect. *Alternata* are phenotypically similar and may reproduce variation between many morpho-species. These characteristics may be deceptive in the identification of these small-spored *Alternaria* species and would require stringent identification at the DNA level [21].

The multigene phylogeny with the combined dataset showed a maximum likelihood (ML) topology in identifying the small-spored *A. alternata* fungus. All 30 *A. alternata* isolates clustered in the *A. alternata* clade sect. *Alternata* in the phylogenetic tree. A previous multi-locus phylogenetic study [19] established the taxonomic conclusions of morphospecies known under *A. alternata* based on the multi-locus phylogenetic analysis. Subsequently, Woudenberg et al. [37] used the same analysis to determine the discrete linages of *Alternaria* spp. in section *A. alternata,* which showed a 97–98% genomic similarity, concluding that species such as *A. tenuissima* (CBS 918.96), *A. mali* (CBS 106.24), *A. malvae*, *A. lini*, *A. citri*, and *A. angustiovoide* did not make discrete groupings, but all are synonymous with *A. alternata* sensu stricto. Based on these most recent classifications, the isolates from South African pecans are thus identified as *A. alternata*.

The single-gene phylogenies showed unclear resolution because of the limited number of informative sites per gene, prompting bias in phylogenetic hypotheses. On the same note, the *A. alternata* subclades were not similar between the four individual gene sequences. These genes evolve at various rates and have different effective ways of discriminating between several phylogenetic scales. For instance, Lawrence et al. [22] reported that *Tef1* and *Rbp2* are slow-evolving genes used to resolve early divergences in *Alternaria*, while *Alt a1* is fast-evolving and can be used to infer evolutionary relationships at lower phylogenetic scales [38]. A combined analysis of all these genes is thus the only approach to identify species delineated by Woudenberg et al. [37].

Even though multiple *Alternaria* spp. have been reported to be associated with black spot disease and seedling blights on different hosts [26], we have only identified *A. alternata* from South African pecans to date. The pathogenicity tests indicated that all the *A. alternata* isolates are potentially pathogenic to cv. Wichita and cv. Ukulinga, with the ability to cause necroses in nuts, leaves, and roots. The inoculated isolates exhibited similar symptoms to those of ABS symptoms in the field, where ABS disease was characterised by the formation of dark, brown-coloured spots that are necrotic in the area of infection [35].

The wounded and unwounded tissues inoculated with the *A. alternata* isolates were significantly different (*p* < 0.001) based on lesion sizes. A possible explanation for this is that *A. alternata* is an opportunistic pathogen that uses wounded sites to invade, damaging the detached nut tissue and thus obtaining nutrients from the host [70]. The artificial inoculation was performed to mimic the mode of attack by wounding, which can either be related to abiotic or biotic factors.

Our findings indicated that Ukulinga cv. were more prone to lesion development than Wichita. This is, however, unusual as observations in the field suggest that Ukulinga is one of the cultivars with good disease resistance and limited symptoms of nutrient deficiencies. For example, Wichita is observed to always show scab symptoms in the eastern parts of South Africa, but not on Ukulinga. This was confirmed with a study of Theron [71], indicating that Wichita was more susceptible to scab than Ukulinga. This highlights the notion that one should not generally accept that, if a cultivar is resistant to one pathogen, it will also be resistant to other pathogens. This indicates that the mechanisms by which pathogens infect pecans, and the factors influencing disease resistance, are not yet fully understood, and further investigation is needed.

No other fungal growth or symptoms were observed that were different from those that were inflicted. This is supported by previous studies on the wounding of plant tissues with *Alternaria* spp. [72]; wounding was shown in this study to be an important disease factor affecting the South African pecan industry. Nonetheless, a previous study [56] also showed that insects can cause wounding on pecans in South Africa, which contributed to the dissemination of fungal pathogens such as *A. alternata*.

All isolates in the present study were identified as *A. alternata* from across South Africa. These isolates originated not only from diseased pecan tissue, but also from healthy material, indicating that the fungus has adopted an endophytic or latent lifestyle [73]. The presence of this ubiquitous pathogen in healthy and resistant pecans over time contributes to the susceptibility of pecan trees [14]. This is also an indication that stress factors could play a role in the overall health of pecan trees [35]. This has been shown in other plants or crops, such cotton (*Gossypium hirsutum*) leaf senescence in China [74], cruciferous vegetables in Europe [68], and more recently on pear (*Pyrus communis*) in the Netherlands [72]. Control and management are challenging as *A. alternata* can only be eradicated systemically [75], and even harvested pecan nuts-in-shucks and kernels that appear healthy at this point can subsequently start showing symptoms due to endophytes such as *A. alternata* during storage and transport [76]. Although the impact of ABS on nut development would be a worthwhile endeavour for future study, it seems plausible that the severe infection of *A. alternata* that causes ABS could potentially have an effect on the poor filling of developing nuts.

The seedling inoculations provided evidence that *A. alternata* isolates are pathogenic to roots, eventually causing the death of the inoculated pecan seedlings. To date, there is no information available on *Alternaria* causing wilting of pecan seedlings or matured trees. Nayyar et al. [77] in Pakistan revealed that healthy seedlings of sesame (*Sesamum indicum*) were seriously affected by *A. alternata* spore suspensions. It was previously shown that the production of AAL toxins produced by *A. alternata* inhibited the growth of roots and shoots of plants such as *Xanthium italicum* [78], *Sida hermaphrodita* [79] and *Triticum* spp. [80]. In the current study, discolorations and necroses were observed on the roots. It is not clear whether the seedlings died only due to the development of necrosis, or whether AAL toxins could have played a role. However, given the severity of the disease, the impact of *A. alternata* on pecan wilt could be a concern to pecan producers.

The occurrence of *A. alternata* in pecan production areas in South Africa, due to its ubiquitous nature, indicates that this fungus is likely already present when young pecan trees from nurseries are planted in the field. Previously, pecan producers often purchased young trees from non-certified pecan nurseries, resulting in poor-quality plants [7]. However, the establishment of certified nurseries required stringent disease management practices such as the application of fungicides, physical screening of seedling plants, use of sterile water, and drip irrigation instead of overhead irrigation [81], although these strategies could only be useful for a limited period. The introduction of long-term solutions such as the production of superior pathogen-free material and resistant pecan cultivars could prove to be challenging but necessary options.

## 5. Conclusions

We investigated *Alternaria* fungi associated with black spot disease and seedling wilt of pecan (*Carya illinoinensis*) in South Africa by molecular and pathogenicity assays to better understand the dominant causal species and potential virulent effects on the host. The multigene DNA sequence phylogeny was successful in identifying *A. alternata* at the species level, and the study further confirmed that this fungus is the cause of ABS and seedling wilt. In the future, screening for large-scale fungal association from more pecan areas is needed to mitigate the fungal diversity. Further studies on the control of ABS disease and seedling wilt under field conditions are warranted to determine the management strategy for the pecan industry in South Africa.

## Figures and Tables

**Table 1:** Details of primers, sequences, annealing temperatures, amplicon lengths, and references used in this study.

Gene	Primer	Sequence (5′-3′)	Tm (°C)	Amplicon Length (Approx. No. bp)	Reference
*Alt a1*	Alt-F	ATG CAG TTC ACC ACC ATC GC	63.3 °C	980	[42]
	Alt-R	ACG AGG GTG AYG TAG GCG TC			
*Gapdh*	Gpd1	CAA CGG CTT CGG TCG CAT TG	58.2 °C	860	[43]
	Gpd2	GCC AAG CAG TTG GTT GTG			
*Rpb2*	Rpb2-5F	GAY GAY MGW GAT CAY TTY GG	58.2 °C	1020	[44]
	Rpb2-7cR	CCC ATR GCT TGY TTR CCC AT			
*Tef1*	EF1	ATG GGT AAG GAR GAC AAG AC	61.3 °C	720	[45]
	EF2	GGA RGT ACC AGT SAT CAT G			

*Alt a1*: Alternaria major allergen, *Gapdh*: Glyceraldehyde-3-phosphate dehydrogenase, *Rpb2*: RNA polymerase II 2nd largest subunit, *Tef1*: Translation elongation factor 1-α, Bp: base pair.

**Table 2:** Seedling wilt tests of *Alternaria alternata* isolates on pecan seedlings.

*Alternaria alternata* Isolate	Disease Severity (DSI) ^a^	Rating Scale ^b^	Aggressiveness Level ^c^
CGJM3006	80.0 ^b^	3	A
CGJM3103	80.0 ^b^	3	A
CGJM3078	90.0 ^a^	3	A
CGJM3136	80.0 ^b^	3	A
CGJM3137	50.0 ^c^	2	M
CGJM3142	90.0 ^a^	3	A
Control	0.00 ^d^	0	-

**^a^** Values with same letter are not significantly different based on Fisher’s LSD test (*p* < 0.05). **^b^** 0 = no wilting symptoms, 1 = 25% wilting, 2 = 50% wilting and 3 = 100% wilting and plant death 56-days post inoculation. **^c^** Degree of disease severity: (-) no symptom, aggressive (A), moderately aggressive (M).

## Data Availability

The datasets generated during and/or analysed during the present study are available from the corresponding author on reasonable request.

## Supplementary Materials

The following supporting information can be downloaded at: https://www.mdpi.com/article/10.3390/pathogens12050672/s1, Table S1: Sampling details of **Alternaria *alternata* isolates recovered from pecans grown in major production locations in South Africa; Table S2: Three-way ANOVA summary table of the detached nut assay of pecan showing their interaction effects of**Alternaria *alternata* isolate, treatment (wounded and unwounded), and cultivar (Wichita and Ukulinga) data sets; Table S3: Three-way ANOVA summary table of the detached leaf assay of pecan showing their interaction effects of**Alternaria *alternata* isolate and treatment (wounded and unwounded) of Wichita cultivar data set; Figure S1A: Maximum likelihood tree of *Alternaria *section *Alternaria *based on Alt a 1 gene DNA sequences. Bootstrap support values are indicated on the branch nodes. The identified* A. alternata* isolates (highlighted in blue) clustered within the* A. alternata* species complex. No geographical locality and symptom classification amongst the* A. alternata* isolates; Figure S1B: Maximum likelihood tree of *Alternaria *section *Alternaria *based on Gapdh gene DNA sequences. Bootstrap support values are indicated on the branch nodes. The identified* A. alternata* isolates (highlighted in blue) clustered within the* A. alternata* species complex. No geographical locality and symptom classification amongst the* A. alternata* isolates; Figure S1C: Maximum likelihood tree of *Alternaria *section *Alternaria *based on Rpb2 gene DNA sequences. Bootstrap support values are indicated on the branch nodes. The identified* A. alternata* isolates (highlighted in blue) clustered within the* A. alternata* species complex. No geographical locality and symptom classification amongst the* A. alternata* isolates; Figure S1D: Maximum likelihood tree of *Alternaria *section *Alternaria *based on Tef1 gene DNA sequences. Bootstrap support values are indicated on the branch nodes. The identified* A. alternata* isolates (highlighted in blue) clustered within the* A. alternata* species complex. No geographical locality and symptom classification amongst the* A. alternata* isolates; Figure S2.**Alternaria *alternata* cultures recovered from treated roots of seedling wilt: (a) CGJM3006, (b) CGJM3013, (c) CGJM3078, (d) CGJM3136, (e) CGJM3137, and (f) CGJM3142.
