# Supplementary material for: Multigene Phylogeny and Pathogenicity Trials Revealed Alternaria alternata as the Causal Agent of Black Spot Disease and Seedling Wilt of Pecan (Carya illinoinensis) in South Africa
Source: Pathogens. 2023 May 2;12(5):672. doi: 10.3390/pathogens12050672 (PMC10223959; doi:10.3390/pathogens12050672)
Supplement: Supplementary file 1 [file pathogens-12-00672-s001.zip › pathogens-2290295-supplementary.pdf]

Supplementary Materials

Table S1. Sampling details of *Alternaria alternata* isolates recovered from pecans grown in major production locations in South Africa

***Alternaria alternata* isolates**	**Location (Town, Province)**	**Coordinates**	**Plant tissue**
CGJM3136	Cullinan, Gauteng	S 25°35'13.6" E 028°33'31.6"	Symptomatic shoots
CGJM3097	Cullinan, Gauteng	S 25°35'13.6" E 028°33'31.6"	Symptomatic leaves
CGJM3102	Modemolle, Limpopo	S 24°40'40.5" E 028°29'55.6"	Symptomatic shoots
CGJM2972	Mookgophong, Limpopo	S 24°25'42.1" E 028°35'41.0"	Symptomatic leaves
CGJM3080	Mookgophong, Limpopo	S 24°25'42.1" E 028°35'41.0"	Non-symptomatic leaves
CGJM3006	Mbombela, Mpumalanga	S 25 26'14.2" E 030 56'01.5"	Symptomatic leaves
CGJM3032	Weenen, KwaZulu-Natal	S 28 51'01.7" E 030 06'01.5"	Symptomatic nuts
CGJM3142	Weenen, KwaZulu-Natal	S 28 51'29.4" E 030 05'21.3"	Symptomatic nuts
CGJM3056	Muden, Kwa-Zulu Natal	S 28 59'27.4" E 030 22'32.4"	Symptomatic nuts
CGJM3095	Pongola, KwaZulu-Natal	S 27 36'25.8" E 031 27'21.3"	Symptomatic shoots
CGJM3078	Cradock, Eastern Cape	S 32 01'42.4" E 025 32'52.7"	Symptomatic shoots
CGJM3089	Cradock, Eastern Cape	S 32 01'42.4" E 025 32'52.7"	Symptomatic nuts
CGJM3121	Cradock, Eastern Cape	S 32 01'42.4" E 025 32'52.7"	Non-symptomatic leaves
CGJM3122	Cradock, Eastern Cape	S 32 01'42.4" E 025 32'52.7"	Non-symptomatic leaves
CGJM3090	Cradock, Eastern Cape	S 32 01'42.4" E 025 32'52.7"	Symptomatic leaves
CGJM2997	Aliwal North, Eastern Cape	S 30 39'49.6" E 026 49'36.7"	Symptomatic nuts
CGJM2966	Aliwal North, Eastern Cape	S 30 39'49.6" E 026 49'36.7"	Symptomatic nuts
CGJM3087	Aliwal North, Eastern Cape	S 30 39'49.6" E 026 49'36.7"	Symptomatic leaves
CGJM3103	Skeerpoort, North West	S 25 47'24.5" E 027 45'53.1"	Non-symptomatic leaves
CGJM3036	Koedoeskop, North West	S 25 13'19.1" E 027 32'02.5"	Symptomatic leaves
CGJM3072	Brits, North West	S 25 32'40.7" E 027 48'56.5"	Symptomatic leaves
CGJM3066	Makwassie, North West	S 27 14'27.4" E 026 09'39.3"	Symptomatic shoots
CGJM2987	Makwassie, North West	S 27 14'27.4" E 026 09'39.3"	Non-symptomatic leaves
CGJM3137	Makwassie, North West	S 27 14'27.4" E 026 09'39.3"	Symptomatic leaves
CGJM2957	Makwassie, North West	S 27 14'27.4" E 026 09'39.3"	Symptomatic leaves
CGJM2991	Coligny, North West	S 26 24'01.0" E 026 12' 32.6"	Symptomatic leaves
CGJM2988	Coligny, North West	S 26 35'05.1" E 026 33'18.8"	Symptomatic leaves
CGJM2969	Coligny, North West	S 26 35'05.1" E 026 33'18.8"	Non-symptomatic leaves
CGJM3060	Syferkuil, North West	S 26 47'35.1" E 026 14'37.9"	Symptomatic leaves
CGJM2981	Skeerpoort, North West	S 25 46'56.9" E 027 45'00.5"	Symptomatic leaves

Table S2. Three-way ANOVA summary table of the detached nut assay of pecan showing their interaction effects of *Alternaria alternata* isolate, treatment (wounded and unwounded), and cultivar (Wichita and Ukulinga) data sets.

Variables[a]	D.f.	Sum Sq.	Mean Sq.	F value	*P*-value
Iso	6	232.7	38.8	7756.0	<0.001 ***
TrT	1	430.2	430.2	86042.9	<0.001 ***
WN-UH	1	216.0	216.0	43200.2	<0.001 ***
Iso:TrT	6	80.7	13.5	2690.2	<0.001 ***
Iso:WN-UN	6	53.6	8.9	1785.8	<0.001 ***
TrT:WN-UN	1	81.0	81.0	16205.4	<0.001 ***
Iso:TrT:WN-UN	6	14.6	2.4	485.5	<0.001 ***
Residuals	56	0.3	0.0		

Significant codes: 0, '***' 0.001 (< 2e-16), '**' 0.01, '*' 0.05, '.' 0.1, ' ' 1.

[a] = Iso (*Alternaria alternata* isolate), TrT (Treatment: wounded and unwounded), WN-UN (Cultivar: Wichita nut and Ukulinga nut).

Table S3. Three-way ANOVA summary table of the detached leaf assay of pecan showing their interaction effects of *Alternaria alternata* isolate and treatment (wounded and unwounded) of Wichita cultivar data set.

Variables[a]	D.f.	Sum Sq.	Mean Sq.	F value	*P*-value
Iso	6	74.59	12.43	10442	<0.001 ***
TrT	1	145.60	145.60	122305	<0.001 ***
Iso:TrT	6	46.60	7.77	6524	<0.001 ***
Residuals	28	0.03	0.00		

Significant codes: 0, '***' 0.001 (< 2e-16), '**' 0.01, '*' 0.05, '.' 0.1, ' ' 1.

[a] = Iso (*Alternaria alternata* isolate), TrT (Treatment: wounded and unwounded).

Alt a 1

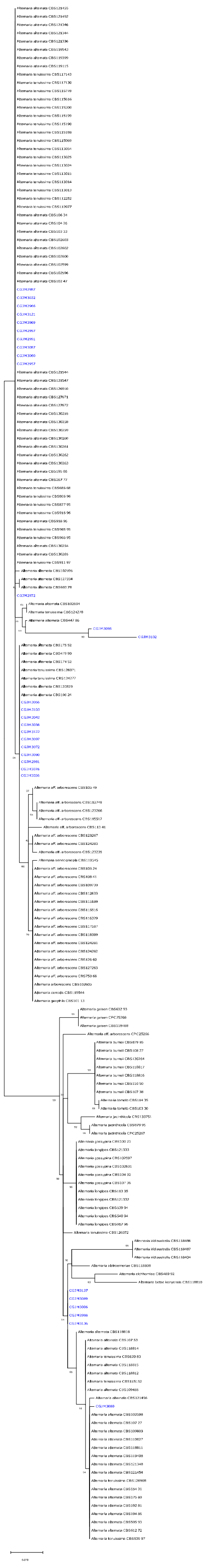

Figure S1A: Maximum likelihood tree of *Alternaria* section *Alternaria* based on *Alt a* 1 gene DNA sequences. Bootstrap support values are indicated on the branch nodes. The identified *A. alternata* isolates (highlighted in blue) clustered within the *A. alternata* species complex. No geographical locality and symptom classification amongst the *A. alternata* isolates.

Gapdh

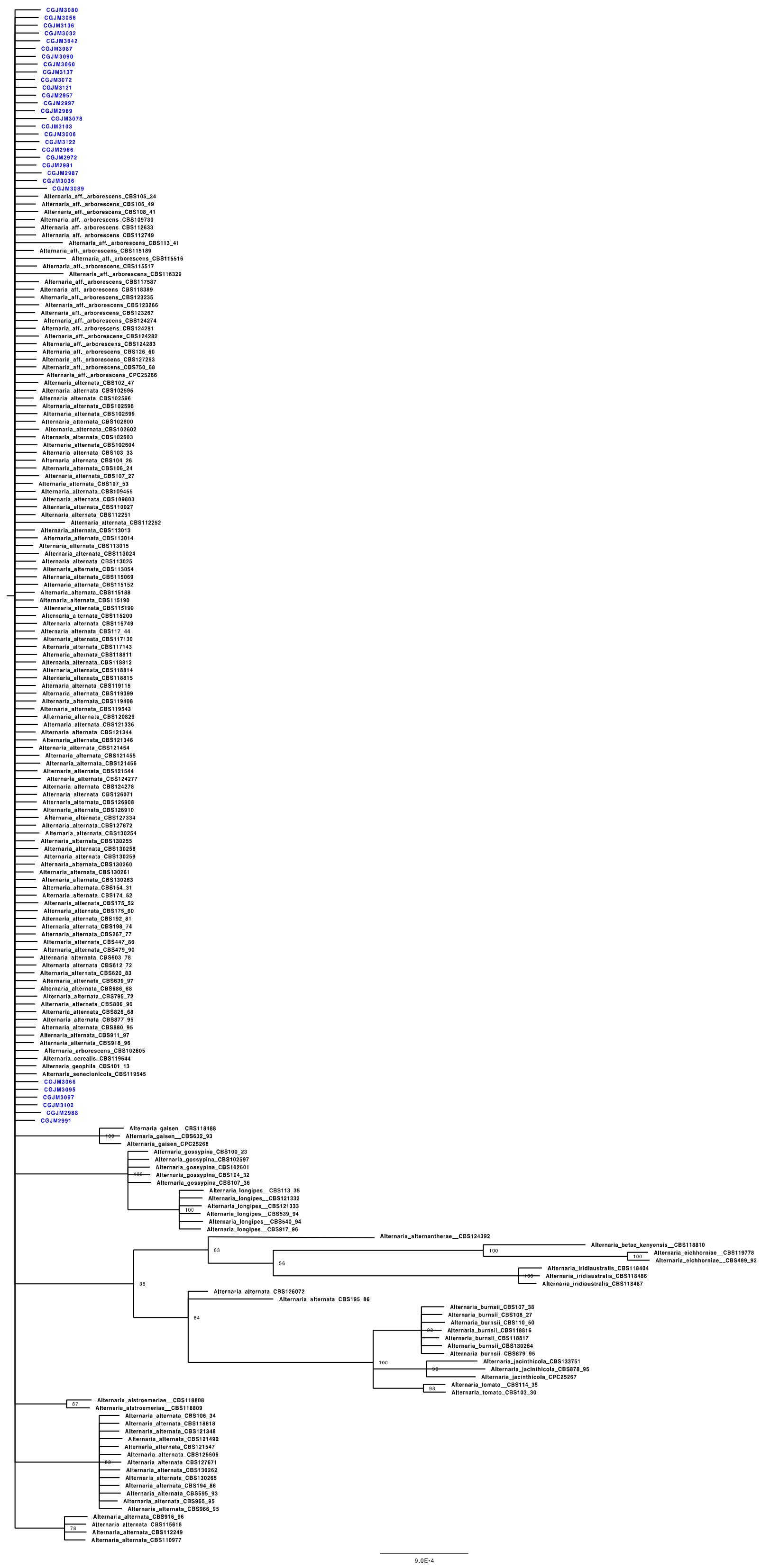

Figure S1B: Maximum likelihood tree of *Alternaria* section *Alternaria* based on *Gapdh* gene DNA sequences. Bootstrap support values are indicated on the branch nodes. The identified *A. alternata* isolates (highlighted in blue) clustered within the *A. alternata* species complex. No geographical locality and symptom classification amongst the *A. alternata* isolates.

Rpb2

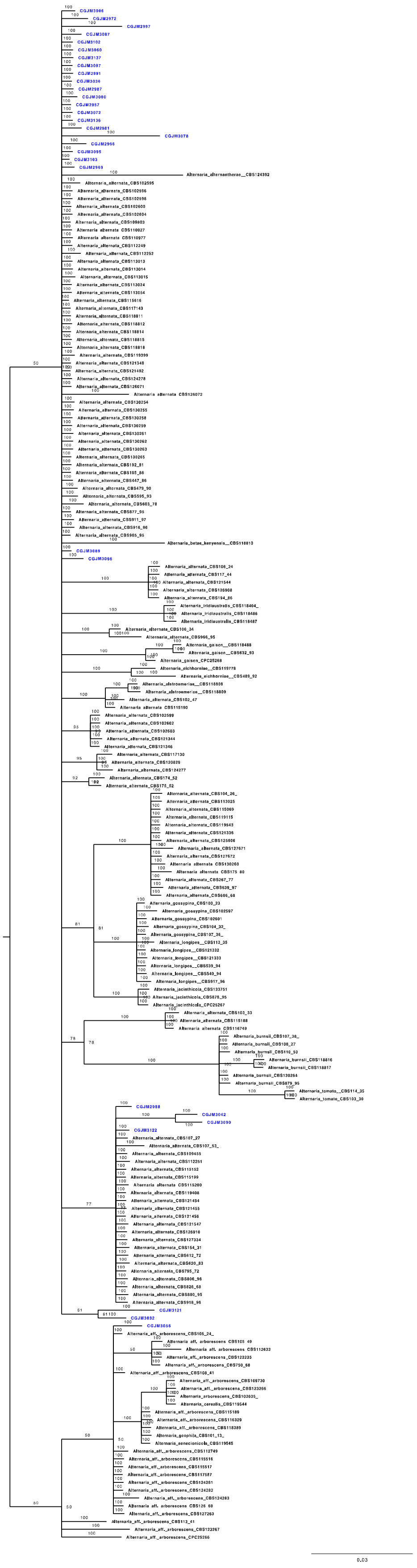

Figure S1C: Maximum likelihood tree of *Alternaria* section *Alternaria* based on *Rpb2* gene DNA sequences. Bootstrap support values are indicated on the branch nodes. The identified *A. alternata* isolates (highlighted in blue) clustered within the *A. alternata* species complex. No geographical locality and symptom classification amongst the *A. alternata* isolates.

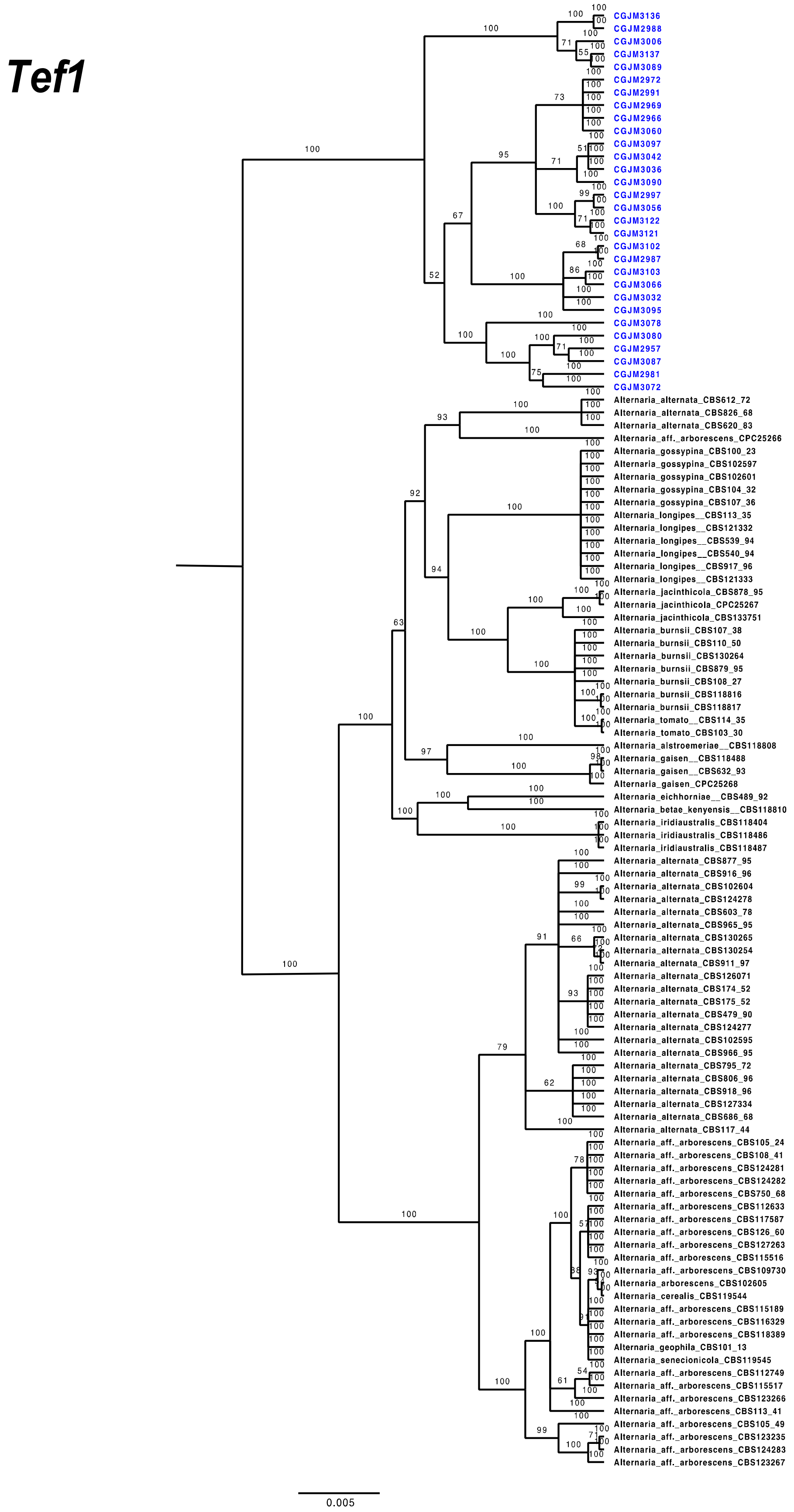

Figure S1D: Maximum likelihood tree of *Alternaria* section *Alternaria* based on *Tef*1 gene DNA sequences. Bootstrap support values are indicated on the branch nodes. The identified *A. alternata* isolates (highlighted in blue) clustered within the *A. alternata* species complex. No geographical locality and symptom classification amongst the *A. alternata* isolates.

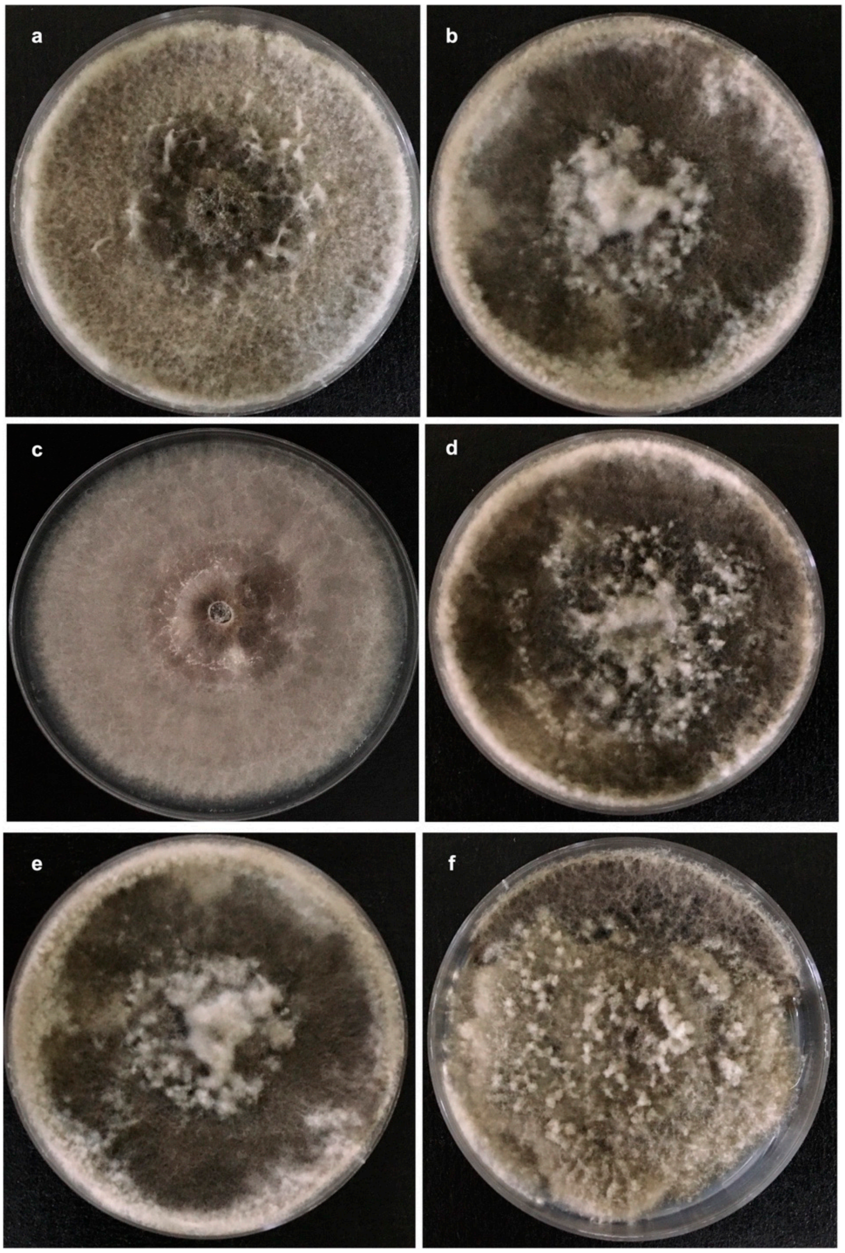

Figure S2. *Alternaria alternata* cultures recovered from treated roots of seedling wilt: **(a)** CGJM3006, **(b)** CGJM3013, **(c)** CGJM3078, **(d)** CGJM3136, **(e)** CGJM3137, and **(f)** CGJM3142.
